# Psychometric properties of the mock interview rating scale for schizophrenia and other serious mental illnesses

**DOI:** 10.3389/fpsyt.2023.1150307

**Published:** 2023-04-27

**Authors:** Matthew J. Smith, Jane K. Burke-Miller, Lindsay A. Bornheimer, Brittany Ross, Morris D. Bell, Susan R. McGurk, Kim T. Mueser, Adrienne Brown, John Prestipino, Nayab Borghani, Karley Nelson, Tovah Lieberman, Nicole J. Pashka, Lisa A. Razzano, Michael A. Kallen

**Affiliations:** ^1^School of Social Work, University of Michigan, Ann Arbor, MI, United States; ^2^Department of Psychiatry, University of Illinois-Chicago, Chicago, IL, United States; ^3^Yale School of Medicine, Department of Psychiatry, New Haven, CT, United States; ^4^Boston University Center for Psychiatric Rehabilitation, Boston, MA, United States; ^5^Thresholds, Chicago, IL, United States; ^6^Feinberg School of Medicine, Northwestern University, Chicago, IL, United States

**Keywords:** schizophrenia, serious mental illness (SMI), employment, job interview skills assessment, psychometric properties

## Abstract

**Background:**

Over the past 10 years, job interview training has emerged as an area of study among adults with schizophrenia and other serious mental illnesses who face significant challenges when navigating job interviews. The field of mental health services research has limited access to assessments of job interview skills with rigorously evaluated psychometric properties.

**Objective:**

We sought to evaluate the initial psychometric properties of a measure assessing job interview skills via role-play performance.

**Methods:**

As part of a randomized controlled trial, 90 adults with schizophrenia or other serious mental illnesses completed a job interview role-play assessment with eight items (and scored using anchors) called the mock interview rating scale (MIRS). A classical test theory analysis was conducted including confirmatory factor analyses, Rasch model analysis and calibration, and differential item functioning; along with inter-rater, internal consistency, and test-retest reliabilities. Pearson correlations were used to evaluate construct, convergent, divergent, criterion, and predictive validity by correlating the MIRS with demographic, clinical, cognitive, work history measures, and employment outcomes.

**Results:**

Our analyses resulted in the removal of a single item (sounding honest) and yielded a unidimensional total score measurement with support for its inter-rater reliability, internal consistency, and test-retest reliability. There was initial support for the construct, convergent, criterion, and predictive validities of the MIRS, as it correlated with measures of social competence, neurocognition, valuing job interview training, and employment outcomes. Meanwhile, the lack of correlations with race, physical health, and substance abuse lent support for divergent validity.

**Conclusion:**

This study presents initial evidence that the seven-item version of the MIRS has acceptable psychometric properties supporting its use to assess job interview skills reliably and validly among adults with schizophrenia and other serious mental illnesses.

**Clinical Trial Registration:**

NCT03049813.

## Introduction

Competitive employment contributes to greater quality of life, physical and mental health, social capital, and reduced poverty for adults with schizophrenia and other serious mental illnesses [e.g., bipolar disorder, major depression (1–3)]. However, only 10–20% of this marginalized and underserved population obtains competitive employment (4). The employment rate increases to as much as 55% among adults with schizophrenia and other serious mental illnesses engaged in evidence-based supported employment such, as the individual placement and support program (5). However, at least 45% of these individuals still face ongoing challenges to securing employment.

The job interview has been identified as a notable barrier to successfully landing a job in the general population (6, 7) and among individuals with schizophrenia and other serious mental illnesses (8). Notably, individuals with schizophrenia and other serious mental illnesses face challenges with their social skills that likely interfere with their ability to effectively navigate conversations such as those that take place during a job interview (9–12). Moreover, the COVID-19 pandemic has resulted in job interviews shifting more frequently to remote meeting platforms where one’s responses to interview questions on a computer screen may be even more challenging for individuals who already have difficulty making real-time social judgments on how to respond to questions (13, 14). Relatedly, a recent study noted that approximately 90% of employed young adults with mental health challenges and other disabilities who engaged in employment services interviewed to obtain their job (15). Moreover, a recent study suggests that approximately 70% of persons with schizophrenia (or other serious mental disorders) who are engaged in supported employment services discussed their interview skills with their employment specialist, and approximately 50% of these individuals completed job interview role-plays with their employment specialist (16). Thus, job interview preparation has emerged as common practice in supported employment programs. The importance of preparing for the job interview is also evident in one study of peer support specialists who believed that their lived experience struggling with job interviews could help them contextualize the value of job interview training for their own clients with schizophrenia or other serious mental illnesses (17).

Given that job interview skills are important to helping people with schizophrenia and other serious mental illnesses get competitive employment (18), there is a need to evaluate more systematic approaches to job skills training in supported employment programs. Relatedly, in order to evaluate the effects of such skills training, there is a need for reliable and valid methods for assessing job interviewing performance. Role play assessments have been used for decades to measure social skills among adults with schizophrenia and other serious mental illnesses and have been shown to be reliable, valid, and sensitive to change following social skills training (19–21). Building on this history, role play assessments of more specific social skills such as job interviewing have emerged in the field. For example, several randomized controlled trials evaluating an internet-delivered job interview training programs recently emerged in lab settings and within a community mental health services agency [e.g., (16, 22, 23)]. Although all of these studies evaluated job interview skills using one job interview role-play assessment [called the mock interview rating scale (MIRS)], which displayed strong inter-rater reliability and sensitivity to change [e.g., (16, 22, 23)], the psychometric properties of this assessment have yet to be evaluated.

Thus, the current study sought to assess the psychometric properties of the MIRS assessment among individuals with schizophrenia or other serious mental illnesses who participated in a randomized controlled trial (RCT) to evaluate *virtual reality job interview training* (VR-JIT); a job interview simulator commercially licensed by SIMmersion LLC^1^ that provides automated scoring and feedback following trainees’ completion of job interview role-plays with a virtual hiring manger (16). To evaluate the structure of items measured in the MIRS assessment, we used published measurement development standards that recommend implementing confirmatory factor analyses along with Rasch model analytics and calibration (24). Thus, we evaluated the reliability of the MIRS via inter-rater reliability, internal consistency, and test-retest reliability.

Further, we sought to evaluate the construct, convergent, divergent, criterion, and predictive validity of the MIRS using correlational analyses. Given that job interview skills fall under the general construct of pragmatic social ability or competence, the relationship between job interview skills and pragmatic social competence reflects a measure of construct validity. Based on job interview and job acquisition frameworks (25–29) and social competence research in schizophrenia (30, 31), the relationship between job interview skills and education, cognitive ability, psychopathology, and perceived utility of job interview training could reasonably serve as measures of convergent validity.

For divergent validity, we hypothesized that age, race, and sex would have no association with interview skills as prior studies did not find age-, race-, or sex-specific differences in work-based social skills (25, 32). Based on the job interview and job acquisition frameworks (25–29), the relationship between job interview skills and employment training or history (e.g., prior vocational training, prior full-time employment, prior part-time employment, and length of absence from the work force) could serve as measures of criterion validity. Meanwhile, the relationship between interview skills (from the IPS-as-usual group at post-test) and acquired competitive employment and number of job offers received by 9-month follow-up (post-randomization) could serve as measures of predictive validity given that prior studies found that post-test MIRS scores predicted receiving offers (18).

## Methods

### Participants

Data were primarily obtained from baseline assessments of participants in an RCT evaluating VR-JIT effectiveness within the individual placement and support (IPS) model of supported employment (16). A total of 90 participants met study inclusion criteria at baseline, were randomized, and were included in the current analysis. Inclusion criteria were: (1) ≥18 years of age; (2) diagnosis of schizophrenia, schizoaffective disorder, bipolar disorder, or major depression via the Structured Clinical Interview for the DSM-V (33); (3) ≥4th-grade reading level, assessed with the Wide Range Achievement Test-V (34); (4) currently unemployed or under-employed (i.e., employed full or part-time but actively seeking new employment); (5) currently job seeking; and (6) willing to be video-recorded for assessments. Exclusion criteria were: (1) another disability or medical illness that could significantly compromise cognition (e.g., traumatic brain injury); (2) documented uncorrected vision or hearing problems that would prevent them from using VR-JIT; and (3) actively suicidal within the past 30 days, assessed using the Columbia-suicide severity rating scale (35). See Smith et al. (16) for more details regarding this RCT.

### Procedures

The study protocol was approved by the Northwestern University (STU00202936) and University of Michigan (HUM00131437) Institutional Review Boards. All procedures were reviewed, approved, and monitored by a Data and Safety Monitoring Board. All study measures were collected or administered by clinical research staff trained and monitored for fidelity. All participants provided their informed consent.

### Measures

#### Job interview skills

Participants completed two interview role-plays during a baseline visit. First, participants reviewed brief postings for eight different part-time jobs (i.e., data entry technician at a university, mail clerk or paralegal at a law firm, medical records clerk at a hospital, inventory manager or stock clerk at a supermarket, sales associate at a home goods store, or reference librarian at a county public library; see Appendix A). Second, participants were given an instruction card that corresponded to each of their selected job scenarios. The instructions read: “(fill in company name and details).” They are interviewing for part-time workers, 25–30 h per week. Starting pay is $15 per hour. You are interviewing for part time work. Third, the participants reviewed their instruction card for 5 min and then engaged in the first role-play. Lastly, participants had up to 5 min to review the scenario for the second role-play, and then completed the second role-play.

The MIRS job interview role-play actors were instructed to play the hiring manager characters as friendly and to ask all 13 of the required job interview questions along with at least three additional questions selected at random from a list of 90 optional questions (see Appendix B). Notably, the first role-play scenarios for two participants were not completed to fidelity as the role-play actors played the role in a stern manner and forgot to ask more than 1/3 of the interview questions. Remaining actor performances met fidelity requirements. The job interview role-plays were video-recorded and coded by raters masked to study condition.

In the current study, we used the first role-play for our primary analyses. Of note, we used the second role-play video for our primary analyses for the two participants identified above where the first role-play actor did not meet fidelity performance standards. The MIRS used an anchoring system to rate eight job interview skills on a five-point scale. The eight skills were developed iteratively. First, the VR-JIT development team reviewed the job interview construct literature (25–29) and drafted a series of targeted skills that were then discussed during three day-long expert panel meetings between the VR-JIT development team and two human resource specialists from local businesses (who routinely conduct job interviews), two employment specialists from a high-fidelity IPS supported employment program, and two clients receiving IPS services (36). The eight skills were finalized by the end of these meetings and then validated after review by 10 persons with serious mental illness engaged in IPS supported employment (36). Prior to the VR-JIT efficacy RCTs, the research team adapted the eight skills into nine items along with their associated anchors. Although the efficacy trials used nine items, one item focused on “negotiation skills” was removed from the MIRS as the field has shifted away from “negotiating” until after a job is offered. Thus, the MIRS resulted in the following eight items to assess job interview skills: comfort level during the interview, being a hard worker, sounding easy to work with, sharing things in a positive way, sounding interested in the position, sounding honest, sounding professional, and overall rapport [Appendix C (23)].

#### Participant demographics and baseline work history

Each participant completed a brief survey about their background (e.g., age, sex, race, and education) and work history (e.g., prior full-time employment, prior part-time employment, and length of time working); whether they received vocational services prior to IPS (0 = “no” or 1 = “yes”), and their total days in IPS at baseline (computed using IPS start date and date of baseline visit). Participants were characterized as recent IPS enrollees (i.e., fewer than 90 days in IPS) or IPS nonresponders [i.e., engaged in IPS for 90 days or more without obtaining competitive employment or obtaining work and getting fired (37)].

In addition, we surveyed participants on their perceptions of the utility of job interview training using a seven-item subscale (1 = “not at all true” to 7 = “very true”; *α* = 0.76) from the standardized intrinsic motivation inventory (IMI) (38), plus two stand-alone items. The IMI items focused on perceiving the value or usefulness of an activity (e.g., I believe practicing job interview role-plays could be of some value to me). Two standalone items used the same scaling as the IMI and asked: (1) I understand the purpose of practicing job interview role-plays; and (2) practicing job interview role-plays requires me to focus and concentrate.

#### Clinical characteristics

Participants completed the physical health component (six items; normed scores 0–100) of the 12-item short-form health survey (39). The participants’ psychiatric diagnoses were determined via the administration of the Structured Clinical Interview for the DSM-5 (33). Current psychopathology was assessed with the brief psychiatric rating scale (40, 41). A single composite score represented four domains: (1) thought disturbance, (2) anergia, (3) depressed affect, and (4) disorganization (40, 41). Participants completed the 10-item alcohol use disorders identification test (AUDIT) (42); and the 10-item drug abuse screening test (DAST) (43). We computed summed total scores for the AUDIT and the DAST for analyses, with higher scores reflecting greater unhealthy substance use.

#### Cognitive characteristics

We used the MATRICS consensus cognitive battery (MCCB) (44); and generated individual *T*-scores for: (1) processing speed, (2) attention/vigilance, (3) verbal working memory, (4) verbal learning, (5) visual learning, (6) reasoning and problem-solving, and (7) social cognition (45).

#### Social competence

Participants completed the social skills performance assessment (SSPA) (10); at pre-test and post-test; this is a widely used general test of social competence by measuring conversational skills. The SSPA includes two brief (3 min) role plays in which participants engage in a conversation with an unknown confederate who plays the role of a “new neighbor (NN)” or a “landlord (LL).” The role plays were video-recorded at the pre-test visits, and were rated using an anchoring system. The NN role play (*α* = 0.79) was scored using eight items with a five-point scale (e.g., 5 = “very interested” to 1 = “very disinterested”), while the LL role-play (*α* = 0.88) was scored using nine items with a five-point scale (e.g., 5 = “very focused” to 1 = “very unfocused”). The item-level means were computed for each role play and then averaged for a single social competence score at pre-test. Five primary raters trained to administer the SSPA using the SSPA training protocol (10). Raters jointly scored approximately 10% of all videos to prevent drift (ICC = 0.97).

#### Employment outcomes

Participants completed employment follow-up interviews at post-test (approximately 3 months after randomization) and monthly after post-test for the remainder of the 9-month post-randomization follow-up period. Participants self-reported how many job interviews they completed, how many job offers they received, and whether a competitive job was obtained. A competitive job was defined as located in an integrated community setting where participants were paid at least minimum wage and the position was not set aside for a person with a disability. The competitive employment outcomes were verified using a combination of self-reports, IPS employment records, and reports from employment specialists.

### Missing data

Twenty-three participants had never participated in job interview training and did not answer two items from the IMI that required them to previously engage in job interview training. Multiple imputation methods were used to generate imputed values for these missing items (46). Two standalone items assessed whether one perceived job interview training as requiring focus and whether one performed job interviews well after completing job interview training. The same aforementioned 23 participants did not complete these items; we did not impute values for them. Data were missing for the measures of attention/vigilance (*n* = 6), verbal working memory (*n* = 1), and social competence (*n* = 1). We correlated the MIRS with the missingness of each variable and all were non-significant (all *p* > 0.06). Notably, we analyzed the data without imputation for the variables where the data were missing.

### Psychometric analyses

To evaluate the psychometric properties of the eight-item MIRS, we followed research guidelines recommending an initial evaluation of content validity for clinician-reported assessments (47). The current study utilized state-of-the-science psychometric analyses in our measure assessment to determine the most parsimonious set of items that would yield a valid and reliable unidimensional measure of the underlying latent construct of job interview skills using the MIRS in a population of adults living with SMI. Based on the PROMIS framework (20) and previously implemented by our team (48) and others (49, 50), our comprehensive analytic process applied classical test theory analyses (CTT) including, assessments of item quality and scale dimensionality [confirmatory factor analyses (CFA)] complemented by item response theory (IRT) Rasch model analytics (24). Concurrently, we computed inter-rater and scale reliabilities. The validity of the resulting unidimensional MIRS measure was further assessed by examining construct, convergent, divergent, criterion and predictive relationships with established measures.

#### Item quality

Items needed to have acceptable Pearson *r* item-rest score correlations [≥0.40; (51)]. An item-rest correlation [i.e., the correlation between a single item’s scores and the summed scores of a measure’s other items excluding the single item of interest (the “rest” of the measure’s items)] provides a test of item construct validity. For an item to be considered monotonic, evidence must be provided demonstrating that, as an individual item’s score increases, the mean of the “rest” summed scores must also increase (i.e., the item score status should generally reflect measure score status).

#### Confirmatory factor analysis

Confirmatory factor analysis was performed in Stata [version 17 (52)] and Mplus (53) to confirm item set unidimensionality (54–56). Items with low factor loadings [<0.50 (57)]; or demonstrating local dependence [residual correlation >0.20; correlated error modification index (MI) ≥ 100] were considered as candidates for exclusion (54–56). Unidimensionality was considered confirmed if the following overall model fit criteria were met: comparative fit index (CFI) ≥0.95, Tucker–Lewis index (TLI) ≥0.95, root mean square error of approximation (RMSEA) <0.10, and standardized root mean residual (SRMR) <0.08 (58–60). The weighted least squares: mean and variance adjusted (WLSMV) (61); estimator was utilized. Items were considered multivariate normal.

If our CFA model did not fit our proposed overall model fit criteria and instead suggested possible multi-dimensionality, we conducted a confirmatory bifactor analysis (CBFA) to diagnose that multi-dimensionality’s potential impact (55, 62). We determined if (a) analyzed items were essentially unidimensional (general factor *omega-H* >0.80) and (b) the model’s general factor had a majority of reliable variance attributable to it (*omega-H-to-omega* ratio >0.50), supporting use of the MIRS (56, 63). We then combined evidence from CFA and CBFA modeling to establish MIRS’s essential unidimensionality, which is a requirement for IRT/Rasch.

#### Rasch analysis

We used the common-threshold Andrich rating scale model (RSM; one set of estimated location thresholds for all items). The analysis was performed in Winsteps [version 3.1.2 (64)] to estimate item parameters for a unidimensional item set (65, 66). We examined items for significant misfit to the RSM [i.e., items having mean-square infit or outfit values exceeding the 0.6–1.4 “acceptable fit” range (67)].

#### Differential item functioning

Differential item functioning (DIF) was examined to identify potential item bias, such as items that might unfairly advantage or disadvantage individuals in a given population subgroup. DIF analyses were performed in Winsteps [version 3.1.2 (64)]. The following DIF factors were investigated: IPS status (non-responder vs. recent IPS enrollees), work history (any competitive employment in the past 2 years vs. none), education (some college or higher vs. high school or less), diagnosis (schizophrenia spectrum vs. other disorder), and sex-assigned-at-birth (male vs. female). We considered items to have impactful DIF if they met the following criteria: (a) having a statistically significant (*p* ≤ 0.05) group-specific item parameter difference and a DIF contrast (i.e., difference in between-group item difficulties) effect size ≥0.64 per item tested (68).

#### Rasch item calibration

With our final item set solution for the MIRS, we calibrated items (i.e., estimated their parameters) using the RSM and established a norm-referenced (i.e., person-centered) *T*-score metric (*M* = 50; SD = 10). Item calibrations were then available for person scoring and subsequent reliability and validity assessments.

#### Score distribution characteristics

For the standardized total measure, we obtained the following: minimum and maximum observed scores, the score distribution mean, SD, median, skewness, kurtosis, and percent of participants with the minimum or maximum possible score. The potential floor or ceiling effect were also considered; we specified acceptable floor and ceiling effects as ≤20% of respondents with minimum or maximum possible scores (69, 70). For skewness and excess kurtosis, values from −1.0 to +1.0 are indicative of a score distribution’s essential normality (71).

#### Inter-rater reliability

The principal investigator (PI) trained seven role-play raters using 10 gold standard mock job interview role-play videos. Raters scored all videos, and then the PI and raters discussed each rating for all 10 videos to come to a consensus on the gold standard rating. During the study, raters were expected to score approximately 10% of all videos that in turn would be evaluated for inter-rater reliability via intraclass correlation coefficient analysis.

##### Internal consistency

We assessed the reliability of the final group of MIRS items via Cronbach’s alpha for measures of internal consistency (including McDonald’s omega). We defined “excellent,” “good,” and “acceptable” reliability as follows: “excellent” criterion = reliability ≥0.90; “good” criterion = reliability ≥0.80 to <0.90; and “acceptable” criterion = reliability ≥0.70 to <0.80.

##### Test-retest reliability

We assessed test-retest reliability by correlating the MIRS pre-test variable with the MIRS post-test variable among the participants in the RCT who were randomized to IPS services-as-usual. Although approximately 70% of the IPS services-as-usual group talked with their employment specialist about interviewing and approximately 50% performed job interview role-plays with their employment specialists, this amount of training did not translate to improved job interview skills between pre-and post-test (16).

#### Construct and convergent validities

Pearson correlations were obtained to examine the associations between the MIRS *T*-score and the measures of social competence, sex, years of education, social cognition, neurocognition, psychopathology, and perceptions of job interview training. Our hypotheses were that rated interview skills would positively correlate with social competence (for construct validity) and with being female, social cognition, neurocognition, and perceiving job interview training as valuable (for convergent validity). Meanwhile, job interview skills would negatively correlate with psychopathology (for convergent validity). In our validity analyses, correlations should be >0.3 in absolute value and statistically significant (72, 73). In regard to the validity coefficient analysis, we took note of the factors known to affect the strength of construct and convergent validity coefficients, including scale and criterion scale reliability, skewness, range restriction, timing, and method variance. In particular, the method variance is of note in that self-report and external rater assessments will be less strongly correlated than homogenous method assessments (74).

#### Divergent, criterion, and predictive validities

For divergent, criterion, and predictive validities, we performed Pearson and point-biserial correlations. For divergent validity, we correlated the baseline MIRS *T*-scores with race group (% black, indigenous, and persons of color), physical health, and alcohol or drug misuse. For criterion validity, we correlated the baseline MIRS *T*-scores with employment history (collected at baseline) that included ever having had full-time (or part-time) employment, duration of last full-time job, and having received a job offer for a part-time job in the past six months. We note that criterion validity coefficients rarely exceed 0.3–0.4 in absolute value due to factors previously noted to affect convergent validity coefficients (74), and that scales with moderate correlations with their criteria provide meaningful predictive information (75). For predictive validity, we correlated the post-test MIRS *T*-scores with employment outcomes experienced by the IPS-services-as-usual group (total jobs offered and obtaining competitive employment) collected between randomization and 9-month post-randomization. Given prior studies of the MIRS found that post-test MIRS scores predicted a greater likelihood of receiving job offers (18), a one-tailed test was used for these two correlations. Notably, we chose the IPS services-as-usual group since the parent RCT found that the IPS + VR-JIT group significantly improved their MIRS rating between pre-and post-test, were more likely to get jobs, and trended toward getting their jobs sooner than the IPS services-as-usual group (16). Thus, we wanted to avoid including any data from the IPS + VR-JIT group as the use of VR-JIT could bias the pattern of MIRS results.

#### Score conversion tables

A preliminary raw summed score-to-*T*-score conversion or “lookup” table was created (see Appendix A), which provides an accessible alternative to the need to employ a Rasch RSM anchored-parameter computer program for scoring.

#### Sample size requirements

For our factor analytic work, CFAs require a minimum of *n* = 5 cases per observed variable, particularly when modeling a single latent variable with multiple indicators (76). Thus, our sample size is adequate. Recommendations for Rasch RSM-based analyses suggest a minimum *N* = 50 participants are needed to establish stable item parameters (67), while DIF analyses associated with RSM estimation can be performed, provided there are ~30 participants per subgroup (64). Thus, we determined the current study’s sample size to be appropriate for these analyses.

## Results

### Participant demographics and mean scores on assessments

A descriptive summary of participant demographic, clinical, and cognitive characteristics and work history is presented in Table 1 (*N* = 90). A descriptive summary of the additional variables used to help establish validity of the measures is presented in Table 2.

**Table 1:** Participant background, clinical, and work history characteristics (*n* = 90).

	Mean (SD) or %	Range
Age, *M* (SD)	45.65 (12.84)	23.41–68.68
Biological sex (% male)	56.75	–
Race		
Black or African American (%)	65.60%	–
White (%)	25.60%	–
Latinx (%)	7.80%	–
More than one race (%)	1.00%	–
Years of education, *M* (SD)	12.48 (2.02)	8.00–18.00
Physical health, *M* (SD)	45.53 (10.69)	15.72–62.71
Primary mental health diagnosis		
Schizophrenia spectrum disorder (% yes)	61.10%	–
Bipolar disorder (no psychotic features; % yes)	16.70%	–
Major depressive disorder (no psychotic features; % yes)	22.20%	–
Psychopathology, *M* (SD)	22.74 (6.38)	16.00–46.00
AUDIT, *M* (SD)	4.12 (6.06)	0.00–31.00
DAST, *M* (SD)	0.64 (1.48)	0.00–6.00
Employment history^a^^,^^b^		
Received vocational training prior to IPS enrollment	25.60%	–
Ever had full-time job (*n* = 87; % yes)	67.80%	–
Longest at a full-time job (months; *n* = 61)	50.45 (51.59)	2.00–300.00
Ever had part-time job (% yes)	82.20%	–
Received offer for part-time job in past 6 months (*n* = 28; % yes)	53.60%	–

AUDIT, alcohol use disorders identification test; DAST, drug abuse screening test; IPS, individual placement and support.

a Only *n* = 12 participants reported whether or not they received an offer for a full-time job in the past 6 months.

b The employment history interview in this study did not ask about the longest a part-time job was held.

**Table 2:** Descriptive statistics of social, cognitive, behavioral, and VR-JIT engagement and performance variables (*n* = 90).

	Mean (SD)	Range
Social ability		
Social competence (baseline), *M* (SD) (*n* = 89)	3.76 (0.63)	1.75–4.83
Social cognition, *M* (SD)	35.94 (14.88)	10.00–84.00
Neurocognition		
Processing speed, *M* (SD)	30.76 (13.71)	−8.00–69.00
Attention/vigilance, *M* (SD) (*n* = 84)	34.95 (13.97)	2.00–69.00
Working memory, *M* (SD) (*n* = 89)	32.12 (12.95)	4.00–63.00
Verbal learning, *M* (SD)	36.27 (8.72)	21.00–67.00
Visual learning, *M* (SD)	32.02 (11.78)	9.00–56.00
Perceptions of job interview training		
has value, *M* (SD)	6.37 (0.75)	4.14–7.00
requires focus, *M* (SD) (*n* = 67)	6.39 (1.02)	4.00–7.00
understands the purpose of practicing, *M* (SD) (*n* = 89)	6.18 (1.49)	1.00–7.00

### Psychometric analyses

Examination of item quality using item-rest correlations found Item 6 (“sound honest”) to have below-threshold correlation with the remaining seven items combined (*r* = 0.27). Item 1 (“comfort level”) had the next lowest correlation with remaining items combined at *r* = 0.45, and all other items had item-rest correlations between 0.53 and 0.79.

The initial CFA confirmed that Item 6 was not a strong fit for the underlying dimension, with a less than minimally acceptable standardized factor loading of 0.31 and three of four overall fit indexes not meeting recommended threshold criteria: CFI = 0.96; TLI = 0.94; RMSEA = 0.13; and SRMR = 0.08. Note that local item dependence did not appear as a modeling issue, as no residual correlation was >0.20, nor was any correlated error MI≥ 100. Removing Item 6 “sound honest” resulted in a unidimensional model with all item factor loadings >0.50. Of the seven remaining items, Item 1 “comfort level” had the lowest factor loading but still above the minimum threshold of 0.50 (0.60), with other item loadings ranging from 0.65 (Item 4) to 0.95 (Item 9), supporting the measure’s construct validity and the presence of a single underlying construct. Note that, again, local item dependence did not appear as a modeling issue (no residual correlation >0.20; no correlated error MI≥ 100). However, overall fit indices did not meet threshold criteria for good model fit of the one-factor solution (CFI = 0.96; TLI = 0.94; RMSEA = 0.16; and SRMR = 0.08) (77). We, therefore, conducted a CBFA and determined the MIRS seven-item set was essentially unidimensional: general factor *omega-H* = 0.89; *omega-H-to-omega* ratio = 0.94, i.e., the general factor had 94% of reliable variance attributable to it.

The results of our initial eight-item Rasch model showed good overall item reliability (0.93), person reliability (0.81), item fit (0.99 infit/1.0 outfit), and person fit (1.0 infit/1.0 outfit). However, Item 6 (“sound honest”) had infit and outfit statistics exceeding the threshold of 1.4 (infit = 2.01 and outfit = 1.97). Excluding Item 6, there was still good overall item reliability (0.90), and improved person reliability (0.84), and good overall item and person fit (0.99 infit/1.0 outfit).

After excluding Item 6, the DIF analyses did not yield any significant results with groups defined by IPS responder status, recent work history, education, psychiatric diagnosis, and sex-assigned-at-birth. Both classical test theory and item response theory assessments supported removing one item (Item 6) to yield a parsimonious unidimensional measure. Final content of the seven-item measure is shown in Table 3.

**Table 3:** Final MIRS content.

Item 1. Comfort level
Item 2. Hard worker
Item 3. Easy to work with
Item 4. Sounds positive
Item 5. Sounds interested
Item 6. Sounds professional
Item 7. Overall rapport

MIRS, mock interview rating scale.

#### Score distribution characteristics

Observed the seven-item MIRS *T*-scores (norm referenced from Rasch analysis) ranged from 21.8 to 77.5 (*M* = 50, SD = 10), with possible scores of 9.2–90.2 (see conversion table in Appendix C). Skewness and kurtosis values were −0.09 and 0.32, indicated an essentially normal score distribution. There were no observed floor or ceiling effects (see Table 4).

**Table 4:** MIRS *T*-score descriptive statistics (*n* = 90).

	MIRS *T*-score
Mean	50.0
Median	51.4
Standard deviation	10.0
Skewness	−0.093
Standard error of skewness	0.254
Kurtosis	0.323
Standard error of kurtosis	0.503
Min	21.8
Max	77.5

MIRS, mock interview rating scale.

#### Reliability

##### Interrater reliability

Seven raters scored all 306 study videos (across pre-and post-test). To establish interrater reliability, four raters scored the same 10% of videos (ICC = 0.93). Three raters scored the same 3.8% of videos, as they departed the project before completion: rater 5 (ICC = 0.99), rater 6 (ICC = 0.98), and rater 7 (ICC = 0.91).

##### Internal consistency

The Cronbach’s alpha for the final seven-item MIRS measure (after removing item #6) was good (0.85) as was McDonald’s omega reliability (0.86); these metrics would not have been increased with the elimination of any single item.

##### Test-retest reliability

Among the IPS services-as-usual group in the parent RCT, the seven-item MIRS *T*-score at pre-test had a strong correlation (*r* = 0.82) with the seven-item MIRS *T*-score at post-test (*n* = 32; four participants in this group did not complete a post-test role play).

#### Validity

##### Construct and convergent validities

The following measures were significantly correlated with the MIRS (Table 5) and met the minimal threshold of 0.30: social competence (*r* = 0.48, *p* < 0.001), processing speed (*r* = 0.36, *p* < 0.001), verbal working memory (*r* = 0.32, *p* = 0.002), reasoning and problem solving (*r* = 0.33, *p* = 0.002), perceiving job interview training as valuable (*r* = 0.30, *p* = 0.004), job interview training requires focus (*r* = 0.34, *p* = 0.005), and understanding the purpose of job interview training (*r* = 0.33, *p* = 0.002). The MIRS was significantly (*p* < 0.05) related in the hypothesized direction (but did not meet the 0.30 threshold) for social cognition (*r* = 0.28) and attention/vigilance (*r* = 0.29). Meanwhile, verbal learning and visual learning were in the hypothesized direction but were not significant (*p* > 0.08). Although psychopathology was negatively related to the MIRS (as hypothesized), this relationship did not meet the 0.30 threshold and was non-significant (*p* = 0.064).

**Table 5:** Validity correlations (*n* = 90).

	MIRS *T*-score	
	*r*	*p*
Construct validity		
Social competence (*n* = 89)	0.46	<0.001
Convergent validity		
Sex	0.06	0.579
Social cognition	0.28	0.007
Processing speed	0.36	<0.001
Attention/vigilance (*n* = 84)	0.29	0.008
Verbal working memory (*n* = 89)	0.32	0.002
Verbal learning	0.17	0.119
Visual learning	0.18	0.084
Reasoning and problem solving	0.33	0.002
Psychopathology	−0.20	0.064
Job interview training has value	0.32	0.002
Job interview training requires focus (*n* = 67)	0.34	0.005
Understands the purpose of practicing job interview training (*n* = 89)	0.33	0.002
Divergent validity		
Race (% BIPOC)	−0.05	0.670
Education (years)	<−0.01	0.967
Physical health	−0.11	0.291
Alcohol misuse	−0.08	0.472
Drug misuse	0.07	0.507
Criterion validity		
Employment history^a^^,^^b^		
Ever had full-time job (*n* = 87)	0.16	0.124
Longest at a full-time job (months; *n* = 61)	0.30	0.019
Ever had part-time job	0.05	0.636
Received offer for part-time job in past 6 months (*n* = 28)	0.33	0.088
Predictive validity^c^^,^^d^		
Employment outcomes (IPS-as-usual)		
Total job offers received by nine-month follow-up (*n* = 31)	0.35	0.026
Competitive employment accepted (yes or no, *n* = 32)	0.15	0.210

MIRS, mock interview rating scale; BIPOC, black, indigenous, and persons of color.

a Only *n* = 12 participants reported whether or not they received an offer for a full-time job in the past 6 months. The Pearson correlation was not reported due to the small *n*.

b The employment history interview in this study did not ask about the longest a part-time job was held.

c Predictive validity was assessed using the MIRS data collected at post-test from the services as usual group to avoid potential bias or noise that could be introduced from the targeted virtual reality job interview training group.

d Predictive validity measures were analyzed using a one-tailed test based on prior research reporting post-test MIRS scores predicted receiving job offers (18).

##### Divergent, criterion, and predictive validities

As displayed in Table 5, the following measures were not significantly correlated with the MIRS: race, physical health, alcohol misuse, and drug misuse (all *p* > 0.10). Regarding criterion validity, the longest a full-time job was held (in months; *r* = 0.30, *p* = 0.019) was significantly correlated with the MIRS and met the threshold magnitude (Table 5), while received a part-time job offer in the past 6 months met the threshold magnitude but was non-significant (*r* = 0.33, *p* = 0.088). The variables of ever having (yes or no) a full-time or part-time job did not meet the 0.30 threshold and were not significant (all *p* > 0.10). Regarding predictive validity results (Table 5), the total number of job offers received (*r* = 0.35, *p* = 0.026) met the 0.30 threshold and was significant; while accepting a competitive job (yes or no) did not (*r* = 0.15, *p* = 0.210).

## Discussion

Although some models of vocational rehabilitation focus on rapid job placement (78), having stronger job interview skills is still a gateway to employment for individuals with schizophrenia and other serious mental illnesses. Notably, training interview skills has been associated with improved access to competitive employment for adults with schizophrenia and other serious mental illnesses in several randomized controlled trials [e.g., (16, 22, 79)]. However, research evaluating job interview interventions has not yet reported whether their job interview role-play measures were psychometrically reliable and provided valid assessments of job interview skills. In addition, the field of vocational rehabilitation lacks a standard assessment of job interview skills, which could lend greater insight into the needs of clients with schizophrenia and other serious mental illnesses as they seek out individualized support to meet their needs. As a result, the current study evaluated the initial psychometric properties of the eight-item MIRS in this population.

We first determined that a single item evaluating “sounding honest” should be eliminated given its below-threshold correlation with the remaining seven items, below threshold factor loadings in the CFA, and infit and outfit statistics that exceeded the acceptable threshold. Subsequently, the study revealed that the seven-item MIRS total score was a unidimensional construct via the use of CTT analyses, CFA and CBFA, Rasch modeling, and calibration analyses. Next, we evaluated the MIRS for potential DIF and did not find the presence of potential item bias in how the MIRS functioned for individuals: (1) with schizophrenia spectrum disorder (compared to those with depression or bipolar disorder), (2) who were competitively employed in the past two years (compared to not competitively employed), (3) recent IPS enrollees (compared to IPS non-responders), (4) with college educations (compared to those with high school or less education), and (5) among males (compared to females). Thus, the MIRS should arguably function similarly among these different subgroups.

The evidence in this study suggests the MIRS is a reliable measure. The MIRS has strong internal consistency reliability via Cronbach’s alpha (*α* = 0.85) that exceeded the standard alpha value (i.e., *α* = 0.70) recommended for measures used in research settings for group-level comparisons (80, 81). Although the MIRS’ internal consistency approached the recommended alpha value for instruments used in applied settings (i.e., *α* = 0.90), it did not meet this threshold (81). In addition, the MIRS had strong inter-rater reliability among four raters (ICC = 0.93) and the test-retest reliability results suggests the MIRS is stable over time. Notably, the MIRS has demonstrated sensitivity to change over time in five efficacy RCTs and two effectiveness RCTs to date (16, 22, 23, 82–85) where significant group-by-time interactions revealed the MIRS value increased over time among groups using virtual job interview training as compared to control or services-as-usual groups where the MIRS value did not change over time.

Regarding validity, our evidence lends initial support for the MIRS’ construct and convergent validities, as several significant correlations met the minimal *r* = 0.30 threshold (72, 73). Specifically, these variables included an independent role-play measure of general social competence for construct validity and multiple markers of neuropsychological function (i.e., processing speed, working memory, reasoning, and problem solving) and the perceived value and utility of job interview training for convergent validity. Notably, measures of social cognition and attention were significantly correlated with the MIRS at *r* = 0.28 or higher, but did not meet the *r* = 0.30 threshold to support convergent validity (72, 73). There were some non-significant correlations in the expected direction with lower magnitudes (e.g., verbal learning, psychopathology). The lack of these relationships could reflect that the ability to learn may be less relevant than other cognitive abilities that are needed when navigating a job interview (e.g., engaging one’s working memory when formulating responses to interview questions). The magnitude of the association between psychopathology and the MIRS was smaller (*r* = −0.20, *p* = 0.064) as compared with prior research examining the relationship between mental health symptomatology and barriers to employment (29). However, the salience of this relationship is noteworthy, given psychopathology was clinically rated and the interview skills were performance-based and rated by content experts masked to group assignment.

Our results revealed that race, physical health, and alcohol and drug misuse did not correlate with the MIRS (25, 32), which suggests the presence of divergent validity, as we did not expect the MIRS to be associated with these constructs. The study results provide mixed support for criterion validity as one variable met the correlation magnitude threshold and obtained significance (i.e., longest held a full-time job). Meanwhile, the variable “having received a part-time job offer in the past six months” met the correlation threshold but did not obtain statistical significance. Other correlations were non-significant with low magnitudes (e.g., ever had a full or part-time job).

With respect to predictive validity, we observed that the post-test MIRS from the IPS-as-usual group was associated with total jobs offered during the 9-month follow-up with a significant correlation above the 0.30 cutoff (72, 73). The correlation with total jobs offered is notably the most salient factor related to job interview skills as this suggests that those with stronger skills were offered more jobs. Moreover, this finding is consistent with a prior mechanistic analysis that the post-test MIRS score predicted receiving a job offer (18). Although the post-test MIRS was not correlated with accepting a competitive job, there are several extraneous factors that could influence whether individuals with schizophrenia or other serious mental illnesses accept a job offer. A few examples include: the position could generate too much income where one’s benefits could be at risk, the job offered may reflect required working hours that do not align with public transportation schedules, or psychiatric symptoms could trigger fear of commitment, success, or socialization (86–90).

### Implications for research and practice

This initial study to evaluate the psychometric properties of the MIRS yielded several positive findings among its structure, reliability, and validity analyses. Thus, future studies may consider using the seven-item version of this assessment to evaluate the efficacy and effectiveness of innovative interventions focused on remediating job interview skills. Moreover, the need for strong job interview skills has likely been increased by the COVID-19 pandemic. Specifically, online job interviews have become common and could be a more challenging platform in which to convey your skills and enthusiasm for obtaining a job. As such, the use of the MIRS could help provide vocational rehabilitation programs serving adults with schizophrenia and other serious mental illnesses with a more standard, evidence-informed means to assess their clients’ job interview skills.

For example, the MIRS could be particularly helpful within the individual placement and support model of supported employment, which emphasizes conducting an initial needs assessment (78, 91, 92). Although IPS has a variety of engagement tools available to providers, they do not yet have a standardized and accepted model of job interview role play. Given that approximately 50% of IPS clients engage in job interview role play training (16), the MIRS could potentially support this process. Thus, the MIRS could further inform and refine individualized services for adults with schizophrenia or other serious mental illnesses, both important aspects of IPS fidelity. Lastly a standardized job interview role play could significantly help ease the burden of training new IPS staff to provide a uniform assessment. Providers are largely left creating their own training resources for staff, and the freely available MIRS would power an amazing data collection tool of the assessment findings of the IPS client base before service begins.

### Limitations and future directions

Results must be considered within the context of the study limitations. First, the parent RCT was not designed to evaluate the psychometric properties of the MIRS. Therefore, the scope of variables to evaluate the MIRS’ validity was limited. Second, support for the MIRS’ criterion validity was limited, as only two (of five) variables were significant and met the 0.30 threshold. Notably, all baseline employment history variables were collected through self-reported methods that required participants to rely on their memories, which may be limited, as individuals with schizophrenia and other serious mental illnesses have cognitive impairments that interfere with recalling personal histories (93, 94). Thus, future research may consider using a timeline follow-back method to help participants remember their employment histories. Third, job interview skills may be influenced by a number of factors among individuals with schizophrenia and other serious mental illnesses that were not examined in the present study. For example, we did not have a physiological measure of job interview anxiety, which is a commonly experienced state that disrupts one’s interview skills (95–97). Fourth, interview skills are like any other skill that diminishes when not in use. Therefore, participants may have had stronger interview skills at the time they obtained jobs in their past, but, if they have not exercised those skills recently then that relationship (between baseline MIRS and employment history) may have diminished. Fifth, our evaluation of predictive validity only included the IPS-as-usual group and had limited statistical power, which should be considered when interpreting the results. Sixth, several participants did not complete all of their monthly follow-ups to report their employment outcomes. Thus, missing data on the number of job interviews completed and job offers received outcomes were conservatively coded as negative employment outcomes, which may have resulted in an underestimate of the correlation between the MIRS and employment outcome. Lastly, given that the eliminated item assessing “sounding honest” was identified through stakeholder input as critical for the job interview performance (36), future research may consider reconceptualizing how this item is scored prior to re-evaluating it for potential fit with the MIRS.

### Conclusion

Job interviews are particularly challenging for individuals with schizophrenia and other serious mental illnesses, and the field lacks a psychometrically-evaluated assessment of job interview skills. This study yielded a reliable seven-item measure of job interview skills [i.e., the mock interview rating scale (MIRS)] after removing a single item (sounding honest) that was a poor fit. The MIRS was found to meet several markers of construct, convergent, and divergent validities. Although some markers of criterion and predictive validities of the MIRS were observed, the strength of these result were more limited. Overall, this initial evaluation suggests the MIRS could serve as a suitable assessment in research as well as an approach to determine the strength of one’s interview skills among individuals with schizophrenia or other serious mental illnesses who are engaged in vocational rehabilitation services.

## Data availability statement

The original contributions presented in the study are included in the article/Supplementary material; further inquiries can be directed to the corresponding author.

## Ethics statement

The Northwestern University Institutional Review Board and the University of Michigan Institutional Review Board reviewed and approved all human subjects research procedures. All study participants provided their written informed consent to participate in this study.

## Author contributions

MS, JB-M, LB, MB, SM, KM, LR, and MK contributed to conception and design of the study. BR organized the database. JB-M performed the statistical analysis. MS wrote the first draft of the manuscript. MS, JB-M, LB, BR, MB, SM, KM, AB, JP, NB, KN, TL, NP, LR, and MK contributed to writing sections of the manuscript. All authors contributed to the article and approved the submitted version.

## Funding

This project was supported by funding from the National Institute of Mental Health (R01MH110524; PI: MS).

## Conflict of interest

The University of Michigan, School of Social Work, and MS will receive royalties on sales of an adapted version of a virtual reality job interview training tool that will focus on meeting the needs of autistic transition age youth. The data reported in this manuscript were collected as part a trial evaluating the original version of virtual reality job interview training where the University of Michigan, School of Social Work, and MS will not receive royalties. AB, JP, NB, KN, TL, NP, and LR were employed by Thresholds.

The remaining authors declare that the research was conducted in the absence of any commercial or financial relationships that could be construed as a potential conflict of interest.

## Publisher’s note

All claims expressed in this article are solely those of the authors and do not necessarily represent those of their affiliated organizations, or those of the publisher, the editors and the reviewers. Any product that may be evaluated in this article, or claim that may be made by its manufacturer, is not guaranteed or endorsed by the publisher.

## Footnotes

^1^ www.simmersion.com

## References

[1] Marwaha S Johnson S. Schizophrenia and employment—a review. Soc Psychiatry Psychiatr Epidemiol. (2004) 39:337–49. doi: 10.1007/s00127-004-0762-4 15133589

[2] Ruesch P Graf J Meyer PC Rossler W Hell D. Occupation, social support and quality of life in persons with schizophrenic or affective disorders. Soc Psychiatry Psychiatr Epidemiol. (2004) 39:686–94. doi: 10.1007/s00127-004-0812-y 15672288

[3] Bouwmans C de Sonneville C Mulder CL Hakkaart-van RL. Employment and the associated impact on quality of life in people diagnosed with schizophrenia. Neuropsychiatr Dis Treat. (2015) 11:2125–42. doi: 10.2147/ndt.S83546, PMID: 26316759 PMC4547637

[4] Drake RE Whitley R. Recovery and severe mental illness: description and analysis. Can J Psychiatr. (2014) 59:236–42. doi: 10.1177/070674371405900502, PMID: 25007276 PMC4079142

[5] Frederick DE Vander Weele TJ. Supported employment: meta-analysis and review of randomized controlled trials of individual placement and support. PLoS One. (2019) 14:e0212208. doi: 10.1371/journal.pone.0212208, PMID: 30785954 PMC6382127

[6] Macan T. The employment interview: a review of current studies and directions for future research. Pers Psychol. (2009) 19:203–18. doi: 10.1016/j.hrmr.2009.03.006

[7] Posthuma RA Morgeson FP Campion MA. Beyond employment interview validity: a comprehensive narrative review of recent research and trends over time. Pers Psychol. (2002) 55:1–81. doi: 10.1111/j.1744-6570.2002.tb00103.x

[8] Solinski S Jackson HJ Bell RC. Prediction of employability in schizophrenic patients. Schizophr Res. (1992) 7:141–8. doi: 10.1016/0920-9964(92)90044-6 1515375

[9] Dickinson D Bellack AS Gold JM. Social/communication skills, cognition, and vocational functioning in schizophrenia. Schizophr Bull. (2007) 33:1213–20. doi: 10.1093/schbul/sbl067, PMID: 17164469 PMC2632341

[10] Patterson TL Moscona S McKibbin CL Davidson K Jeste DV. Social skills performance assessment among older patients with schizophrenia. Schizophr Res. (2001) 48:351–60. doi: 10.1016/s0920-9964(00)00109-2, PMID: 11295387

[11] Bellack AS Morrison RL Wixted JT Mueser KT. An analysis of social competence in schizophrenia. Br J Psychiatry J Ment Sci. (1990) 156:809–18. doi: 10.1192/bjp.156.6.809 2207511

[12] Gilbert E Marwaha S. Predictors of employment in bipolar disorder: a systematic review. J Affect Disord. (2013) 145:156–64. doi: 10.1016/j.jad.2012.07.009, PMID: 22877965

[13] Chandratre S Soman A. Preparing for the interviewing process during Coronavirus disease-19 pandemic: virtual interviewing experiences of applicants and interviewers, a systematic review. PLoS One. (2020) 15:e0243415. doi: 10.1371/journal.pone.0243415, PMID: 33284848 PMC7721161

[14] Oliffe JL Kelly MT Gonzalez Montaner G Yu Ko WF. Zoom interviews: benefits and concessions. Int J Qual Methods. (2021) 20:160940692110535. doi: 10.1177/16094069211053522

[15] Smith MJ Sherwood K Blajeski S Ross B Smith JD Jordan N. Job interview and vocational outcomes among transition-age youth receiving special education pre-employment transition services. Intellect Dev Disab. (2021) 59:405–21. doi: 10.1352/1934-9556-59.5.405, PMID: 34551103 PMC10732084

[16] Smith MJ Smith JD Blajeski S Ross B Jordan N Bell MD. An RCT of virtual reality job interview training for individuals with serious mental illness in IPS supported employment. Psychiatr Serv. (2022) 73:1027–38. doi: 10.1176/appi.ps.202100516, PMID: 35172592 PMC9661916

[17] Üstel P Smith MJ Blajeski S Johnson JM Butler VG Nicolia-Adkins J. Acceptability and feasibility of peer specialist-delivered virtual reality job interview training for individuals with serious mental illness: a qualitative study. J Technol Hum Serv. (2021) 39:219–31. doi: 10.1080/15228835.2021.1915924 37139353 PMC10153604

[18] Smith MJ Smith JD Fleming MF Jordan N Brown CH Humm L. Mechanism of action for obtaining job offers with virtual reality job interview training. Psychiatr Serv. (2017) 68:747–50. doi: 10.1176/appi.ps.201600217, PMID: 28292223 PMC5495604

[19] Bellack AS Hersen M Lamparski D. Role-play tests for assessing social skills: are they valid? Are they useful? J Consult Clin Psychol. (1979) 47:335–42. doi: 10.1037/0022-006X.47.2.335, PMID: 469081

[20] Bellack AS Morrison RL Mueser KT Wade JH Sayers SL. Role play for assessing the social competence of psychiatric patients. Psychol Assess. (1990) 2:248–55. doi: 10.1037/1040-3590.2.3.248

[21] Bellack AS Mueser KT Gingerich S Agresta J. Social Skills Training for Schizophrenia: A Step-by-Step Guide, vol. p. xi. 2nd ed New York: Guilford Press (2004). 337 p.

[22] Smith MJ Fleming MF Wright MA Roberts AG Humm LB Olsen D. Virtual reality job interview training and 6-month employment outcomes for individuals with schizophrenia seeking employment. Schizophr Res. (2015) 166:86–91. doi: 10.1016/j.schres.2015.05.022, PMID: 26032567 PMC4512907

[23] Smith MJ Ginger EJ Wright M Wright K Boteler Humm L Olsen D. Virtual reality job interview training for individuals with psychiatric disabilities. J Nerv Ment Dis. (2014) 202:659–67. doi: 10.1097/NMD.0000000000000187, PMID: 25099298 PMC4149584

[24] PROMIS®. Instrument development and validation. (2013). Available at: https://staging.healthmeasures.net/images/PROMIS/PROMISStandards_Vers2.0_Final.pdf

[25] Huffcutt AI. An empirical review of the employment interview construct literature. Int J Sel Assess. (2011) 19:62–81. doi: 10.1111/j.1468-2389.2010.00535.x

[26] Huffcutt AI Conway JM Roth PL Stone NJ. Identification and meta-analytic assessment of psychological constructs measured in employment interviews. J Appl Psychol. (2001) 86:897–913. doi: 10.1037/0021-9010.86.5.897, PMID: 11596806

[27] Constantin KL Powell DM McCarthy JM. Expanding conceptual understanding of interview anxiety and performance: integrating cognitive, behavioral, and physiological features. Int J Sel Assess. (2021) 29:234–52. doi: 10.1111/ijsa.12326

[28] Huffcutt AI Roth PL McDaniel MA. A meta-analytic investigation of cognitive ability in employment interview evaluations: moderating characteristics and implications for incremental validity. J Appl Psychol. (1996) 81:459–73. doi: 10.1037/0021-9010.81.5.459

[29] Corbiere M Zaniboni S Lecomte T Bond G Gilles PY Lesage A. Job acquisition for people with severe mental illness enrolled in supported employment programs: a theoretically grounded empirical study. J Occup Rehabil. (2011) 21:342–54. doi: 10.1007/s10926-011-9315-3, PMID: 21656251

[30] Mueser KT Bellack AS Morrison RL Wade JH. Gender, social competence, and symptomatology in schizophrenia: a longitudinal analysis. J Abnorm Psychol. (1990) 99:138–47. doi: 10.1037//0021-843x.99.2.138, PMID: 2348007

[31] Hooley JM. Social factors in schizophrenia. Curr Dir Psychol Sci. (2010) 19:238–42. doi: 10.1177/0963721410377597

[32] Huffcutt AI Roth PL. Racial group differences in employment interview evaluations. J Appl Psychol. (1998) 83:179–89. doi: 10.1037/0021-9010.83.2.179

[33] First MB Williams JBW Karg RS Spitzer RL. Structured Clinical Interview for DSM-5 Disorders: Research Version (SCID-5-RV). Washington, DC: American Psychiatric Press (2015).

[34] Wilkinson GS Robertson GJ. Wide Range Achievement Test (WRAT-5). 5th ed. Bloomington, MN: Pearson, Inc. (2017).

[35] Posner KBG Stanley B Brent DA Yershova KV Oquendo MA Currier GW. The Columbia-suicide severity rating scale: initial validity and internal consistency findings from three multisite studies with adolescents and adults. Am J Psychiatr. (2011) 68:1266–77. doi: 10.1176/appi.ajp.2011.10111704 PMC3893686 22193671

[36] Bell MD Weinstein A. Simulated job interview skill training for people with psychiatric disability: feasibility and tolerability of virtual reality training. Schizophr Bull. (2011) 37:S91–7. doi: 10.1093/schbul/sbr061, PMID: 21860052 PMC3160120

[37] McGurk SR Mueser KT Xie H Welsh J Kaiser S Drake RE. Cognitive enhancement treatment for people with mental illness who do not respond to supported employment: a randomized controlled trial. Am J Psychiatr. (2015) 172:852–61. doi: 10.1176/appi.ajp.2015.14030374, PMID: 25998278

[38] McAuley E Duncan T Tammen VV. Psychometric properties of the intrinsic motivation inventory in a competitive sport setting: a confirmatory factor analysis. Res Q Exerc Sport. (1989) 60:48–58. doi: 10.1080/02701367.1989.10607413, PMID: 2489825

[39] Ware JE Kosinski M Keller SD. 12-item short form health survey-construction of scales and preliminary tests of reliability and validity. Med Care. (1996) 34:220–33. doi: 10.1097/00005650-199603000-00003 8628042

[40] Lukoff D Nuechterlein K Ventura J. Manual for the expanded brief psychiatric rating scale. Schizophr Bull. (1986) 12:594–602.

[41] Mueser KT Curran PJ McHugo GJ. Factor structure of the brief psychiatric rating scale in schizophrenia. Psychol Assess. (1997) 9:196–204. doi: 10.1037/1040-3590.9.3.196

[42] Saunders JB Aasland OG Babor TF De La Fuente JR Grant M. Development of the alcohol use disorders identification test (audit): who collaborative project on early detection of persons with harmful alcohol consumption-II. Addiction. (1993) 88:791–804. doi: 10.1111/j.1360-0443.1993.tb02093.x, PMID: 8329970

[43] Skinner HA. The drug abuse screening test. Addict Behav. (1982) 7:363–71. doi: 10.1016/0306-4603(82)90005-3 7183189

[44] Nuechterlein KH Green MF Kern RS Baade LE Barch DM Cohen JD. The MATRICS consensus cognitive battery, part 1: test selection, reliability, and validity. Am J Psychiatr. (2008) 165:203–13. doi: 10.1176/appi.ajp.2007.07010042, PMID: 18172019

[45] Mayer JD Salovey P Caruso DR. Mayer–Salovey–Caruso Emotional Intelligence Test (MSCEIT) User’s Manual. Toronto: MHS Publishers (2002).

[46] Rubin DB. Multiple Imputation for Nonresponse in Surveys. Hoboken, NJ: John Wiley & Sons, Inc. (1987).

[47] Powers JH 3rd Patrick DL Walton MK Marquis P Cano S Hobart J. Clinician-reported outcome assessments of treatment benefit: report of the Ispor clinical outcome assessment emerging good practices task force. Value Health. (2017) 20:2–14. doi: 10.1016/j.jval.2016.11.005, PMID: 28212963 PMC5379997

[48] Kallen MA Lai JS Blackwell CK Schuchard JR Forrest CB Wakschlag LS. Measuring Promis^®^ global health in early childhood. J Pediatr Psychol. (2022) 47:523–33. doi: 10.1093/jpepsy/jsac026, PMID: 35552435 PMC9113277

[49] Werb D Richardson C Buxton J Shoveller J Wood E Kerr T. Development of a brief substance use sensation seeking scale: validation and prediction of injection-related behaviors. AIDS Behav. (2015) 19:352–61. doi: 10.1007/s10461-014-0875-z, PMID: 25119056 PMC4450886

[50] Slocum-Gori SL Zumbo BD. Assessing the unidimensionality of psychological scales: using multiple criteria from factor analysis. Soc Indic Res. (2011) 102:443–61. doi: 10.1007/s11205-010-9682-8

[51] Zijlmans EAO Tijmstra J van der Ark LA Sijtsma K. Item-score reliability in empirical-data sets and its relationship with other item indices. Educ Psychol Meas. (2018) 78:998–1020. doi: 10.1177/0013164417728358, PMID: 30542214 PMC6236637

[52] Stata Corp. Stata Statistical Software: Release 17. College Station, TX: Stata Corp LLC (2021).

[53] Muthen LK Muthen BO In: Muthen LK Muthen BO, editors. Mplus User’s Guide (Version 7.4). Los Angeles, CA: (2015)

[54] Cook KF Kallen MA Amtmann D. Having a fit: impact of number of items and distribution of data on traditional criteria for assessing IRT’s unidimensionality assumption. Qual Life Res. (2009) 18:447–60. doi: 10.1007/s11136-009-9464-4, PMID: 19294529 PMC2746381

[55] McDonald RP. Test Theory: A Unified Treatment. New York: Lawrence Erlbaum Associates, Inc. (1999).

[56] Reise SP Morizot J Hays RD. The role of the bifactor model in resolving dimensionality issues in health outcomes measures. Qual Life Res. (2007) 16:19–31. doi: 10.1007/s11136-007-9183-7, PMID: 17479357

[57] Hair JF Jr Black WC Babin BJ Anderson RE. Multivariate Data Analysis. 7th ed New York: Pearson Prentice Hall (2009).

[58] Bentler PM. Comparative fit indexes in structural models. Psychol Bull. (1990) 107:238–46. doi: 10.1037/0033-2909.107.2.238 2320703

[59] Lt H Bentler PM. Cutoff criteria for fit indexes in covariance structure analysis: conventional criteria versus new alternatives. Struct Equ Model Multidiscip J. (1999) 6:1–55. doi: 10.1080/10705519909540118

[60] Kline RB. Principles and Practice of Structural Equation Modeling. New York: Guilford Publications (2015).

[61] Muthen BO. Robust inference using weighted least squares and quadratic estimating equations in latent variable modeling with categorical and continuous outcomes. Psychometrika.

[62] Lai J-S Crane PK Cella D. Factor analysis techniques for assessing sufficient unidimensionality of cancer related fatigue. Qual Life Res. (2006) 15:1179–90. doi: 10.1007/s11136-006-0060-6, PMID: 17001438

[63] Reise SP Scheines R Widaman KF Haviland MG. Multidimensionality and structural coefficient bias in structural equation modeling: a bifactor perspective. Educ Psychol Meas. (2013) 73:5–26. doi: 10.1177/0013164412449831

[64] Linacre JM. Winsteps^®^ Rasch Measurement Computer Program User’s Guide. 4.0.1. Beaverton, Oregon: Winsteps.com (2017).

[65] Andrich D. A rating formulation for ordered response categories. Psychometrika. (1978) 43:561–73. doi: 10.1007/BF02293814

[66] Samejima F. Estimation of latent ability using a response pattern of graded scores. Psychometrika. (1969) 34:1–97. doi: 10.1007/BF03372160

[67] Linacre JM. Sample size and item calibration stability. Rasch Measur Transac. (1994) 7:328.

[68] Peabody MR Wind SA. Exploring the stability of differential item functioning across administrations and critical values using the Rasch separate calibration *t*-test method. Meas Interdiscip Res Perspect. (2019) 17:78–92. doi: 10.1080/15366367.2018.1533782

[69] Andresen EM. Criteria for assessing the tools of disability outcomes research. Arch Phys Med Rehabil. (2000) 81:S15–20. doi: 10.1053/apmr.2000.20619 11128900

[70] Cramer D. Howitt D. (2004). The SAGE Dictionary of Statistics. Thousand Oaks, CA: Sage

[71] Hair JF Jr Hult GTM Ringle CM Sarstedt M. A Primer on Partial Least Squares Structural Equation Modeling (PLS-SEM). Thousand Oaks, CA: Sage Publications (2021).

[72] Pelletier J-F Davidson L Giguère C-É Franck N Bordet J Rowe M. Convergent and concurrent validity between clinical recovery and personal-civic recovery in mental health. J Pers Med. (2020) 10:163. doi: 10.3390/jpm10040163, PMID: 33053639 PMC7712080

[73] Cohen J. Statistical Power Analysis for the Behavioral Sciences. 2nd ed New York: Erlbaum (1988).

[74] Furr RM. Psychometrics: An introduction. Thousand Oaks, CA: SAGE Publications (2021).

[75] Nunnally JC Bernstein IH. Psychometric Theory. New York: McGraw-Hill (1994).

[76] Bentler PM Chou C-P. Practical issues in structural modeling. Sociol Methods Res. (1987) 16:78–117. doi: 10.1177/0049124187016001004

[77] Brown TA. Confirmatory Factor Analysis for Applied Research. 2nd ed New York: Guilford Press (2015).

[78] Becker DR Swanson SJ Reese SL Bond GR McLeman BM. Evidence-Based Supported Employment Fidelity Review Manual. 3rd ed. Lebanon, NH: Westat (2015).

[79] Smith MJ Fleming MF Wright MA Jordan N Humm LB Olsen D. Job offers to individuals with severe mental illness after participation in virtual reality job interview training. Psychiatr Serv. (2015) 66:1173–9. doi: 10.1176/appi.ps.201400504, PMID: 26130002 PMC4630147

[80] Taber KS. The use of Cronbach’s alpha when developing and reporting research instruments in science education. Res Sci Educ. (2018) 48:1273–96. doi: 10.1007/s11165-016-9602-2

[81] Nunnally J. Psychometric Theory. New York, NY: McGraw Hill (1978).

[82] Smith MJ Ginger EJ Wright K Wright MA Taylor JL Humm LB. Virtual reality job interview training in adults with autism Spectrum disorder. J Autism Dev Disord. (2014) 44:2450–63. doi: 10.1007/s10803-014-2113-y, PMID: 24803366 PMC4167908

[83] Smith MJ Boteler Humm L Fleming MF Jordan N Wright MA Ginger EJ. Virtual reality job interview training for veterans with posttraumatic stress disorder. J Vocat Rehabil. (2015) 42:271–9. doi: 10.3233/JVR-150748, PMID: 27721645 PMC5051574

[84] Smith MJ Bell MD Wright MA Humm L Olsen D Fleming MF. Virtual reality job interview training and 6-month employment outcomes for individuals with substance use disorders seeking employment. J Vocat Rehabil. (2016) 44:323–32. doi: 10.3233/JVR-160802, PMID: 31656389 PMC6814393

[85] Smith MJ Parham B Mitchelll J Blajeski S Harrington M Ross B. Virtual reality job interview training for adult offenders receiving prison-based employment services: a randomized controlled feasibility and effectiveness trial. Crim Justice Behav. (2022) 50:272–93. doi: 10.1177/00938548221081447 PMC11178324 38881730

[86] Fyffe DC Lequerica AH Ward-Sutton C Williams NF Sundar V O’Neill J. Understanding persons with disabilities’ reasons for not seeking employment. Rehabil Couns Bull. (2021) 66:3–12. doi: 10.1177/00343552211006773

[87] Audhoe SS Nieuwenhuijsen K Hoving JL Sluiter JK Frings-Dresen MHW. Perspectives of unemployed workers with mental health problems: barriers to and solutions for return to work. Disabil Rehabil. (2018) 40:28–34. doi: 10.1080/09638288.2016.1242170, PMID: 27756177

[88] United States Bureau of Labor Statistics. Persons with a Disability: Barriers to Employment, Types of Assistance, and Other Labor-Related Issues Summary—July 2021. (2022) United States Department of Labor, Bureau of Labor Statistics.

[89] Rosenheck R Leslie D Keefe R McEvoy J Swartz M Perkins D. Barriers to employment for people with schizophrenia. Am J Psychiatr. (2006) 163:411–7. doi: 10.1176/appi.ajp.163.3.411 16513861

[90] Charette-Dussault E Corbiere M. An integrative review of the barriers to job Acquisition for people with severe mental illnesses. J Nerv Ment Dis. (2019) 207:523–37. doi: 10.1097/NMD.0000000000001013, PMID: 31259790

[91] Becker DR Drake RE Bond GR. The IPS supported employment learning collaborative. Psychiatr Rehabil J. (2014) 37:79–85. doi: 10.1037/prj0000044, PMID: 24512479

[92] Bond GR Drake RE. Making the case for IPS supported employment. Admin Pol Ment Health. (2014) 41:69–73. doi: 10.1007/s10488-012-0444-6, PMID: 23161326

[93] Bell M Tsang HW Greig TC Bryson GJ. Neurocognition, social cognition, perceived social discomfort, and vocational outcomes in schizophrenia. Schizophr Bull. (2009) 35:738–47. doi: 10.1093/schbul/sbm169, PMID: 18245058 PMC2696363

[94] Bearden CE Glahn DC Monkul ES Barrett J Najt P Villarreal V. Patterns of memory impairment in bipolar disorder and unipolar major depression. Psychiatry Res. (2006) 142:139–50. doi: 10.1016/j.psychres.2005.08.010 16631256

[95] McCarthy J Goffin R. Measuring job interview anxiety: beyond weak knees and sweaty palms. Pers Psychol. (2004) 57:607–37. doi: 10.1111/j.1744-6570.2004.00002.x

[96] Feiler AR Powell DM. Behavioral expression of job interview anxiety. J Bus Psychol. (2016) 31:155–71. doi: 10.1007/s10869-015-9403-z

[97] Powell DM Stanley DJ Brown KN. Meta-analysis of the relation between interview anxiety and interview performance. Can J Behav Sci. (2018) 50:195–207. doi: 10.1037/cbs0000108

