# Supplementary material for: Psychometric properties of the mock interview rating scale for schizophrenia and other serious mental illnesses
Source: Front Psychiatry. 2023 Apr 27;14:1150307. doi: 10.3389/fpsyt.2023.1150307 (PMC10172658; doi:10.3389/fpsyt.2023.1150307)
Supplement: Supplementary file 1 [file Data_Sheet_1.PDF]

Appendix A. Mock Interview Rating Scale Job Scenarios

1) Scenario A: The department of Public Health at the local University is running a nation-wide study examining the effects of a newly implemented youth exercise program in elementary schools across the country. We are hiring a team of research assistants to collect and manage our study data. We look forward to building a talented team and have position openings in the following categories: Data Entry Technician, Research Assistant, and Study Coordinator. Position to which they are applying is Data Entry Technician.

2) Scenario B: Goldberg & Jones is a new law firm opening downtown. G&J is a promising new law firm founded by two law students who recently graduated from Kent Law School. G&J is looking for a dedicated staff to ensure the new law firm's success. Goldberg & Jones are seeking to hire individuals for the following positions: Administrative Assistants, Mail Clerks, and Paralegals. Position to which they are applying is Mail Clerk.

3) Scenario C: Lakeview Hospital has provided quality care and treatment for the past 50 years. Lakeview Hospital is currently seeking individuals to add to their dedicated team of employees for the following positions: Medical Records Clerk and Medical Receptionist. Position to which they are applying is Medical Records Clerk.

4) Scenario D: Goldberg & Jones is a new law firm opening downtown. G&J is a promising law firm founded by two law students who recently graduated from Kent Law School. G&J is looking for a dedicated staff to ensure the new law firm's success. Goldberg & Jones are seeking to hire individuals for the following positions: Administrative Assistants, Mail Clerks, and Paralegals. Position to which they are applying is Paralegal.

5) Scenario E: Stop & Shop is the second largest, nation-wide supermarket chain. We are opening a new store in your neighborhood to sell food and household goods. We look forward to building a talented team and have position openings in the following categories: Customer Service, Cashier, Stock Clerk and Inventory. Position to which they are applying is Inventory Manager.

6) Scenario F: At T&C Home Goods, we are committed to quality - both in the value of our product line and in the talent employ. We offer a personalized work environment to hundreds of dedicated associates, with all the perks of a multi-million-dollar company. We offer an extensive selection of home décor and building supplies. Position to which they are applying is Sales Associate.

7) Scenario G: Stop & Shop is the second largest, nation-wide supermarket chain. We are opening a new store in your neighborhood to sell food and household goods. We look forward to building a talented team and have position openings in the following categories: Customer Service, Cashier, Stock Clerk and Inventory. Position to which they are applying is Stock Clerk.

8) Scenario H: The County Public Library enables researchers, students, teachers, and others to have access to the records of the state's past while preserving irreplaceable historical materials for future generations. The sizeable collection of books, journals, manuscripts, newspapers, maps, and prints, available to the public makes it the most frequented library in the state. Position to which they are applying is Reference Librarian

Appendix B: Mock Interview Rating Scale Job Interview Role Play Fidelity Checklist

General reminders: The interview questions you ask don't need to come in any particular order. Instead, there should be a natural flow so feel free to jump around or let the participant guide the conversation.

The questions in red MUST be asked. The rest of the questions you can ask if you have time available. Checkmark all the questions you ask. Interview should take approximately 30 minutes.

Actor Initials: _______

Circle which job scenario you are role-playing:

1 2 3 4 5 6 7 8

Objective 1: Negotiation skills

- ☐ Do you have any questions?

Objective 2: Presenting as a hard worker

- ☐ Why is it important to be on-time?
- ☐ Give me an example of a time when you went above and beyond the call of duty

Objective 3: Sounding easy to work with

- ☐ Can you tell me about a time you had a conflict with a peer or a supervisor and how you resolved it?
- ☐ How do you get along with others when you're working on a team project?

Objective 4: Sharing things in a positive way

- ☐ I'd like to know more about your work history. What kind of jobs have you held in the past?
- ☐ Why'd you leave your last position?

Objective 5: Sounding honest

- ☐ If I were your supervisor and asked you do to something that you disagreed with, what would you do?
- ☐ If you saw a coworker stealing, how would you respond?

Objective 6: Sounding interested

- ☐ If you were offered this job, how long do you see yourself working here?
- ☐ What interests you most about this position?

Objectives 7: Sounding professional

- ☐ Would you have trouble abiding by a zero tolerance alcohol and drug policy?

Miscellaneous

- ☐ What are your strengths and weaknesses?

Objective 1: Continued

- ☐ *Before we move on, is there anything I can answer for you?*
- ☐ *I want to make sure I give you a chance to talk too. Do you have any questions?*
- ☐ *I always ask candidates if they need any accommodations in order to perform this job. Will you?*
- ☐ *Standard work hours are Monday – Friday, 9-5 and occasional weekends. Will this work for you?*
- ☐ *Are you aware of our hours of operation?*
- ☐ *Are you looking for full or part-time work?*

Follow up responses:

- ☐ *Why do you need Thursdays off?*
- ☐ *I don't set schedules. If you're offered the job, you'll have to discuss that with your supervisor.*
- ☐ *Usually schedules are discussed with supervisors after a job offer has been made. But since you bring it up, it usually isn't a problem.*
- ☐ *If you're offered the job, you can bring that up with your supervisor. It shouldn't be an issue, as long as you're willing to be flexible with other days.*
- ☐ *Scheduling varies from supervisor to supervisor, but most plan two weeks of schedule at a time.*
- ☐ *We should be able to accommodate you most weeks, but are you able to work an occasional Thursday?*
- ☐ *Are you sick or something? Why do you need a weekly appointment?*
- ☐ *Thanks for sharing that. It's best to know early on in the scheduling process.*

Objective 2: Continued

- ☐ *Do you have a reliable way to get to work?*
- ☐ *Do you usually show up on-time?*

Objective 3: Continued

- ☐ *Did you get along with people at your last job?*
- ☐ *What were the people at your last job like?*
- ☐ *How would your former coworkers describe you?*
- ☐ *How have you resolved conflicts with coworkers or teammates in the past?*
- ☐ *How do you feel about taking no for an answer?*
- ☐ *What would your previous boss say about you?*
- ☐ *What irritated you most about your previous job?*
- ☐ *Do you work better on a team or by yourself?*
- ☐ *What do you think of your previous boss?*
- ☐ *Who was your favorite manager and why?*
- ☐ *What type of person do you work best with?*
- ☐ *There's a mandatory training program for all new hires. Usually it takes one to two weeks to complete.*
- ☐ *Your first two weeks on the job is considered your training period. You'll learn policies, job duties, and how to perform them.*
- ☐ *I think we have a great training program. It takes one to two weeks. You'll learn scheduling, policies, and a few other things.*

Objective 4: Continued

- ☐ *I noticed on your application that there are gaps in your work history. Can you tell me about that?*
- ☐ *What's the longest you've worked at a company?*
- ☐ *Are you currently employed?*

- ☐ *What was something a supervisor or teacher has told you to improve on in the past?*
- ☐ *What was the worst part of your previous job?*
- ☐ *If you could've changed one thing at your last job, what would it be?*
- ☐ *Tell me about a time that you completed a task and then realized that you'd made a mistake?*
- ☐ *If you completed a task and then realized you'd made a mistake, what would you do?*
- ☐ *Why did you leave your previous position?*

Objective 5: Continued

- ☐ *Have you ever worked on a team where someone wasn't pulling their own weight? How did you handle it?*
- ☐ *If you found out your coworker was doing something against company policy, what would you do?*
- ☐ *What would you do if you caught someone stealing?*
- ☐ *Do you think it's a big deal to clock in a few minutes late or to take breaks that last longer than policy allowed?*
- ☐ *What would you do if a coworker was slacking off?*
- ☐ *How would you handle it if a coworker asked you to help them steal?*
- ☐ *What situation would you consider the most important to report – an employee stealing from customers, the company, or from other employees?*
- ☐ *Tell me about a time you had to follow a policy or rule you didn't agree with.*
- ☐ *Give me an example of a time when you did something wrong. How did you handle it?*

Objective 6: Continued

- ☐ *What are your long-term goals?*
- ☐ *Do you think this job could be satisfying for you long-term?*
- ☐ *What are your career goals?*
- ☐ *Do you think you'll be happy here in ten years?*
- ☐ *Where do you see yourself in ten years?*
- ☐ *How long do you expect to remain employed with this company?*
- ☐ *What are your goals for the next few years? How do you plan to achieve those goals?*

Objective 7: Continued

- ☐ *Have you ever abused drugs or alcohol?*
- ☐ *We have a strict alcohol and drug policy. If you show up to work under the influence, you'll be fired immediately. Would you be able to abide by that policy?*
- ☐ *I know addiction can be tough to deal with. Thanks for sharing that. Do you think this will impact your ability to do the job?*
- ☐ *Are you planning to have children?*

Miscellaneous

- ☐ *Why are you interested in this position?*
- ☐ *What do you know about our company?*
- ☐ *What interests you least about this position?*
- ☐ *Why should I hire you?*
- ☐ *Why do you think you would be a good fit for this position?*
- ☐ *How much experience do you have?*
- ☐ *Do you have any formal education and/or training?*
- ☐ *What have you done outside of education/training to improve yourself?*
- ☐ *What skills can you contribute to this position?*
- ☐ *What skills would you like to use in this position?*
- ☐ What challenges are you looking for in a position?
- ☐ What were your responsibilities in your last position?
- ☐ What was the most rewarding part about your previous position?
- ☐ Have you ever had to go the extra mile to satisfy a customer? What did you do?
- ☐ What's the most difficult decision you've had to make in the last few years?
- ☐ What three character traits would your friends use to describe you?
- ☐ What negative thing would your last boss say about you?
- ☐ What do you like to do for fun?
- ☐ What motivates you?
- ☐ What will/do you miss most about your last job?
- ☐ What type of work environment do you prefer?
- ☐ Describe your ideal job.
- ☐ Describe a time where you had to deliver disappointing/bad news to a customer. What was your approach? How was your message received?
- ☐ Give me an example of a time where you adhered to health/safety requirements even when it wasn't expected/practical to do so.
- ☐ Describe a time in your work experience when you had to think outside the box.
- ☐ Tell me about a time when you had a problem to solve.
- ☐ Give me an example of a time where you had to work on more than one project at once. How did you manage it?
- ☐ Tell me about a time where you created order out of chaos.
- ☐ Describe a team you were on where communication was strong. What made it strong?
- ☐ Give me an example of when you demonstrated resourcefulness.
- ☐ Give some examples where you were able to successfully train someone to perform better.

Appendix C. Mock Interview Rating Scale Anchoring System

Item 1 – Comfort level during interview –

Excellent (score 5)	Average (score 3)	Poor (score 1)
• Excellent interview skills<br>• Enjoying the interview<br>• Displays behavioral comfort	• Minor discomfort<br>• A few signs of anxiety	• Highly anxious<br>• Physical signs of discomfort<br>• Loses train of thought<br>• Taking a long time to respond
Comments:		

5	4	3	2	1

Item 2 – Hard worker –

Excellent	Average	Poor
Comes across dependable. Makes statements about:<br>• Working hard<br>• Paying attention<br>• Asking for more tasks<br>• Doing well working<br>• Making work a priority	Reports behaviors related to both hard work, and unreliability. Or Says they work hard but does not provide examples.	Comes across lazy or unreliable. Makes statements about:<br>• Showing up late<br>• Missing work frequently<br>• Calling in sick, avoiding responsibilities
Comments:		

5	4	3	2	1

Item 3 – Sounding easy to work with –

Excellent	Average	Poor
Seems flexible and likely easy to work with. Makes comments such as:<br>• Gets along with teammates<br>• Helps customers<br>• Takes direction well<br>• Willing to go through training and follow rules/policies	Generally sounds easy to work with, but shares some signs of difficulty. Says they are easy to work with but does not provide examples.	Sounds difficult to work with. Makes statements about:<br>• bad-mouthing coworkers/boss<br>• talks about having conflicts or issues with others<br>• complaining
Comments:		

5	4	3	2	1

Item 4 – Sharing things in a positive way –

<table>
<tr><th colspan="2">Excellent</th><th>Average</th><th colspan="2">Poor</th></tr>
<tr><td colspan="2">• If sharing a story in which they didn’t perform optimally, lessons learned are emphasized
• handles illegal questions well
• Shares past conviction, disability, or other life event in a positive way:

“I didn’t graduate, but I did learn a lot”

“It didn’t relate to this job, but I learned a lot about how to work hard.”</td><td>• Shared positive attributes, but without examples</td><td colspan="2">• handles illegal questions poorly
• shares past conviction, disability, or life event in a negative way:

“I’m a really slow learner”

”Yeah, I took a course, but I didn’t learn anything”</td></tr>
<tr><td colspan="5">Comments:</td></tr>
<tr><td>5</td><td>4</td><td>3</td><td>2</td><td>1</td></tr>
</table>

Item 5 – Sounding interested –

<table>
<tr><th colspan="2">Excellent</th><th>Average</th><th colspan="2">Poor</th></tr>
<tr><td colspan="2">• Several thoughtful, appropriate questions were asked
• Checked next steps for employer and decision date, verified follow-up details
• Affirmed interest in the position
• Cites job description and personal strengths related to job description</td><td>• At least one question was asked
• Cites job description</td><td colspan="2">• No questions were asked
• “any job will do” attitude
• Talking about short-term work
• Questions were inappropriate
• Emphasizes personal preferences like benefits, convenient locale, etc.
• Sounds desperate
• Sounding as though this job would merely be a stepping-stone</td></tr>
<tr><td colspan="5">Comments:</td></tr>
<tr><td>5</td><td>4</td><td>3</td><td>2</td><td>1</td></tr>
</table>

Item 6 – Sounding professional –

<table>
<tr><th colspan="2">Excellent</th><th>Average</th><th colspan="2">Poor</th></tr>
<tr><td colspan="2">• Polite responses (please/thank you)
• Respectful
• Treats interviewer like a supervisor, not a friend.</td><td>Comes across as professional for most of interview, but has a few instances that could be interpreted as unprofessional.</td><td colspan="2">• Oversharing
• Too casual
• Discussing politics, religion, partying, drinking and other inappropriate topics</td></tr>
<tr><td colspan="5">Comments:</td></tr>
<tr><td>5</td><td>4</td><td>3</td><td>2</td><td>1</td></tr>
</table>

Item 7 – Overall rapport –

<table>
<tr><th colspan="2">Excellent</th><th>Average</th><th colspan="2">Poor</th></tr>
<tr><td colspan="2">• Smooth interaction
• Casual and relaxed demeanor
• Engaged in casual conversation with me during the interview
• nodded his/her head while listening to my responses/questions
• smiled frequently, made eye contact
• Enthusiastic without taking over interview</td><td>• Friendly, but lost job-focus
• Small talk was hesitant</td><td colspan="2">• Awkward pauses
• Little eye contact
• Short, brief responses
• Long-Winded, Tangential</td></tr>
<tr><td colspan="5">Comments:</td></tr>
<tr><td>5</td><td>4</td><td>3</td><td>2</td><td>1</td></tr>
</table>

Appendix D. Mock Interview Rating Scale Raw-to-T score conversion table

Raw Summed Score	T Score	T Score SE
7	9.2	10.8
8	16.8	6.2
9	21.8	4.7
10	25.1	4.1
11	27.8	3.8
12	30.3	3.7
13	32.5	3.5
14	34.6	3.5
15	36.7	3.5
16	38.7	3.4
17	40.8	3.5
18	42.8	3.5
19	44.9	3.5
20	47.1	3.5
21	49.2	3.5
22	51.4	3.5
23	53.5	3.5
24	55.7	3.5
25	57.8	3.5
26	59.9	3.5
27	62.1	3.5
28	64.2	3.6
29	66.5	3.6
30	68.8	3.7
31	71.3	3.9
32	74.1	4.2
33	77.5	4.8
34	82.5	6.3
35	90.2	10.8
